# Discovery and characterization of novel lipopeptides produced by *Virgibacillus massiliensis* with biosurfactant and antimicrobial activities

**DOI:** 10.1007/s13205-024-04100-9

**Published:** 2024-10-04

**Authors:** Badiaa Essghaier, Chahnez Naccache, Houda Ben-Miled, Filomena Mottola, Kamel Ben-Mahrez, Maha Mezghani Khemakhem, Lucia Rocco

**Affiliations:** 1 Biochemistry and Biotechnology Laboratory LR01ES05, Faculty of Sciences of Tunis, University of Tunis Elmanar, 2092 Elmanar II, Tunisia; 2 Department of Environmental Biological and Pharmaceutical and Technologies (DiSTABiF), University of Campania L.Vanvitelli-Via Vivaldi, 43-81100 Caserta, Italy

**Keywords:** Biosurfactants, Lipopeptides, *Virgibacillus massiliensis*, Locillomycin, Bacillibactin, WGS

## Abstract

The study aimed to evaluate the biosurfactants (BSs) production by SM-23 strain of *Virgibacillus* identified by phenotypical and WGS analysis as *Virgibacillus massiliensis*. We first demonstrated the lipopeptides production by *Virgibacillus massiliensis* specie and studied their biochemical and molecular analysis as well as their biological potential. The GC–MS analysis indicated that methyl.2-hyroxydodecanoate was the major fatty acid compound with 33.22%. The maximum BSs production was obtained in LB medium supplemented by 1% olive oil (v/v) at 30 °C and 5% NaCl with 1.92 g/l. The obtained results revealed the significant biosurfactants/bioemulsifier potential compared to triton X100 with E24 of 100%, and an emulsification stability SE of 83%. The lipopeptides types were identified by FTIR analysis. A strong antimicrobial action was observed by the produced lipopeptides by the agar diffusion method against *E.coli, K. pneumoniae, S. aureus, Fusarium sp, Alternaria sp,* and *Phytophtora sp.* The complete genome sequencing showed genes involved in the synthesis of multiple compounds identified as amphipathic cyclic lipopeptides such as locillomycin/locillomycin B/locillomycin C and bacillibactin. Our results highlighted significant lipopeptides properties displayed by *V. massiliensis* that can be exploited to develop a novel strategy in the formulation of natural biocidal and fungicidal agents.

**Supplementary Information:**

The online version contains supplementary material available at 10.1007/s13205-024-04100-9.

## Introduction

Surfactants find broad utilization in both household and industrial settings, serving as key components in detergents, emulsifiers, wetting agents, foamers, and dispersants across various industries such as cosmetics, sanitation, food, and petroleum clinics (Roberta et al. 2022). These amphiphilic molecules, featuring both hydrophilic and hydrophobic segments, stand out as the predominant industrial compounds (Mungray and Kumar 2009).

Conversely, surfactants exhibit non-biodegradability, giving rise to secondary pollutants and fostering toxicity, thereby contributing to the emergence of challenging environmental contaminants. Addressing these issues necessitates the adoption of eco-friendly surfactants derived from natural sources (Badmus et al. 2021; Roberta et al. 2022; Collivignarelli et al. 2019; Farias et al. 2021). Biosurfactants, or BSs, represent surfactants sourced from biological origins, synthesized by various microorganisms including yeast, fungi, and bacteria (Plaza and Achal 2021). Noteworthy is the extensive exploration of the Bacillus genus for its production of biosurfactants.

In contrast to synthetic surfactants, BSs exhibit minimal toxicity, superior biodegradability, and versatile physicochemical properties (Zhang et al. 2020; Eleni Drakontis and Samiul Amin 2020). BSs possess the capability to dissolve hydrophobic compounds such as hydrocarbons, lipids, oils, and antibiotics (Moldes et al. 2019).

The distinctive characteristics of BSs are assessed through various parameters including collapse sets, oil droplets, surface tension, emulsification index, and CMC foaming index (Eleni Drakontis and Samiul Amin 2020). Classification based on molecular weight divides BSs into two main groups: low molecular weight (LMW) comprising glycolipids and lipopeptides, and high molecular weight (HMW) encompassing polysaccharides, lipopolysaccharides, proteins, and lipoproteins. These differences in molecular composition significantly influence their metabolic pathways, biological activities, and applications (ErasMuñoz et al. 2022). Notably, lipopeptides and glycolipids are distinguished for their effectiveness compared to HMW counterparts (Chen et al. 2017a, b; Ramani et al. 2012). Among the BSs derived from Bacillus species, cyclic lipopeptides such as lichenysins, bacillomycin, fengycins, and surfactins are notable examples (Giri et al. 2019; Nawazish et al. 2022; Mulligan 2021).

Extremophiles performed unexploited resources for novel bioactive compounds and play a significant role in antimicrobial drug discovery (Sirinivasan et al. 2021). Recently, few studies reported the bactericin production by *Virgibacillus* species, for example *Virgibacillus salexigens* NTN53 which can produce two bactericins NTN-A and NTN-B belonging to Bawcin (Omachi et al. 2021). Bawcin is described in most Bacillus species as well as *Oceanobacillus, Parageobacillus*, and *Virgibacillus* showing robust antimicrobial potential. Nevertheless *Virgibacillus natechei* can synthesize a new peptide that may perform some formulations (Mechri et al. 2019). Previous studies highlighted the beneficial role of *Virgibacillus* species in Agroecology by producing various active compounds, such as *Virgibacillus dokdonensis* (WenChen et al. 2024), *Virgibacillus marismortui* (Essghaier et al. 2011), *Virgibacillus picturae* (Orhan et al. 2023). However, no previous work shared the lipopeptides produced by *Virgibacillus massiliensis*. Several research projects discussed different commercially biosurfactants for pharmaceutical, food, petroleum, agriculture and cosmetic industries, the emerging biotechnological applications of biosurfactants due to their advantages over synthetics ones in terms environment friendly product and safe human health (Leoni et al. 2022).

In previous studies, we delineated the glycolipids synthesized by halophilic bacterial species *Pantoea alhagi* and *Halomonas zhanjiangensis*, showcasing their promising biological attributes (Essghaier et al. 2023a; 2023b). Building upon this foundation, our current investigation focuses on elucidating novel biosurfactants produced by *Virgibacillus* species. Our aims encompass optimizing production processes, ensuring stability, and employing biochemical and molecular methodologies to unveil their chemical structures. To the best of our knowledge, this study represents the maiden report on the biosurfactant production by *Virgibacillus massiliensis*, providing comprehensive biochemical, molecular, and structural characterizations.

## Materials and methods

### Bacterial isolation and identification

#### Bacterial strain isolation and culture conditions

The bacterial strain SM-23 was isolated from Sebkhet El-Meleh in Southern Tunisia. After purification and characterization, the isolate was cultured on Nutrient Agar with 10% NaCl (w/v), at 37 °C for 24 h, for further studies.

#### DNA extraction

The cells from the bacterial culture were harvested and suspended in DNA/RNA Shield buffer (Zyme Research, USA), according to Microbes NG strain submission procedures (Essghaier et al. 2023b).

#### Whole genome sequencing assembly and annotation

Genome sequencing and assembly were conducted based on the method of microbes NG. Genomic DNA libraries were prepared using the Nextera XT library Prep Kit (Illumina, San Diego, USA) with some modifications such as input DNA was increased twofold, and PCR elongation time was increased to 45 s. DNA library was prepared on a Hamilton Microlab STAR automated liquid handling system (Hamilton Bonaduz AG, Switzerland). Libraries were sequenced on an Illumina NovaSeq 6000 (Illumina, San Diego, USA) using a 250pb paired end protocol. Read was trimmed using Trimmomatic version 0.30 (Bolger et al. 2014) with a sliding window quality cutoff of Q15. De novo assembly was performed using SPAdes version 3.7 (Bankevich et al. 2012), and contigs annotated using Prokka 1.11 (Seemann 2014).

#### Genome analysis

To process the taxonomic assignment, the genome sequences were blasted against Refseq Database (1865 high-quality species genomes), then classified based on ANI (average nucleotide identity) values (threshold > 95%). The annotated genes were represented using CGViewer: Circular Genome Viewer based on prokka annotation files. The quality of assessment of contigs and the assessment of assembled genomes were verified based on QUAST (no of contigs, N50, total length, longest sequence, DC content) and CheckM (completeness, quality, contamination), respectively.

### GC–MS analysis of SM-23 strain total fatty acids

The bacterial colonies of strain SM-23 were recuperated, after growing on LB medium at 30 °C for 48 h, in a sterile. The whole fatty acids from bacterial cells were saponified, methylated, and then extracted using the method of MIDI microbial identification system (version 6.0) as described by Sasser (2023). Fatty acid methyl esters were analyzed based on the GC 6890N, network GC system (Agilent technologies, Hewlett Packard) and identified using the TSBA6 database of the identification system (Chen et al. 2017a, b). Twenty-two standards of fatty acids were selected based on their different chain length, geometric structure (cis or trans), and double bond positions (Sigma, Aldrich, Germany) (Essghaier et al. 2023a).

### Biosurfactants detection

#### Hemolytic activity

The hemolysis activity of glycolipids or lipopeptides was examined by the bacterial culture on blood agar plates for 24 h at 37 °C (Rani et al. 2020).

#### Oil spreading activity

500µL of vegetable oil were deposited on the water surface in Petri dishes, and then gently put on 500µL of the cell free culture. The diameter of the clear zone was measured in mm. Triton X100 was used as a positive control (Essghaier et al. 2023a).

#### Emulsification assay

For the emulsification assay, we combined 5 mL of the cell-free culture with 5 mL of olive oil using a vortex mixer for 2 min. The solution was then left at room temperature for 24 h. Measurements were taken at both the initial time (zero time) and after 24 h to ascertain the height of the emulsion layer and the total height of the solution, following the method outlined by Gomaa and El-Mehy (2019). Three replicate analysis was achieved for the assay. The emulsification index was calculated as:

$$\begin{aligned} &{\text{Emulsification}}\;{\text{index}}\,({\text{E}}\,24\left( \% \right) = {\text{emulsion}}\;{\text{height}}\,\left( {{\text{mm}}} \right)\,{\text{after}}\; \, 24{\text{h}}/{\text{total}}\;{\text{height}}\left( {{\text{mm}}} \right) \times 100 \\ &{\text{Emulsification}}\;{\text{ stability}}\,{\text{(SE}}\,\left( \% \right) = {\text{emulsion}}\;{\text{ height}}\,\left( {{\text{mm}}} \right)\,{\text{ after 24h}}/{\text{emulsion }}\;{\text{height }}\left( {{\text{mm}}} \right)\,{\text{at}}\;{\text{ zero}}\;{\text{time}} \times {1}00. \\ \end{aligned}$$

### Biosurfactant production

Different carbon sources (olive oil, corn oil, glucose, fructose) were added separately to LB medium at 0.1%. The BSs production was measured by the estimation of the BSs yield in g/L after extraction. The effect of temperature range (28–30–37–40 °C) and salinity (0–5–10–15% NaCl) was also studied using the optimum carbon sources (olive oil in LB medium).

### Biosurfactant extraction

The acid precipitation method was used for the extraction of biosurfactants as previously described by Essghaier et al. (2023a). The culture broth, incubated for 3 days at 30 °C, was subjected to centrifugation at 12,000 rpm for 20 min. The resulting cell-free culture was acidified to pH 2 using 6N HCl and then refrigerated at 4 °C overnight to induce biosurfactants precipitation. Following another centrifugation at 12,000 rpm for 20 min, the precipitate, comprising crude biosurfactants, was collected, suspended in distilled water, and subsequently lyophilized (Youssef et al. 2005).

### Antimicrobial activity

The agar well diffusion method as previously detailed (Essghaier et al. 2023a; 2011) was employed to assess the potential antibacterial and antifungal effects of the biosurfactants (BSs) against various human bacterial pathogens (*Staphylococcus aureus, Escherichia coli, Serratia marcescens, Klebsiella pneumoniae, Micrococcus lysodeikticus*), as well as fungi, including *Fusarium, Alternaria, Penicillium,* and *Phytophtora*. The pellet BSs was dissolved in ethyl acetate at 2 mg/mL, a volume of 30µL was deposited on the wells.

### FTIR analysis

FTIR spectrum analysis of biosurfactants was carried out on an FTIR Bruker Equinox 55 spectrometer (Bruker Co, Ettlingen, Germany) as previously reported by Essghaier et al. (2023a).

### Analysis of secondary metabolite biosynthetic gene clusters

The antiSMASH framework allows the detection of the biosynthetic gene clusters (BGCs) which contain all the genes required for the biosynthesis of one or more natural products (NPs), also known as specialized or secondary metabolites (Blin et al. 2023). Secondary metabolic biosynthetic gene cluster analysis of the strain SM-23 was carried out by antiSMASH bacterial 7.1.0. AntiSMASH can accurately identify all known secondary metabolic gene clusters when it can use a specific profile hidden Markov models and provide detailed non-ribosomal peptide synthetases (NRPSs) and polyketide synthases (PKSs) functional annotation, and predict the chemical structure of NRPS/PKS products (Blin et al. 2023).

### Statistical analysis

All the screening experiments were performed in triplicate and the mean values were subjected to statistical analyses using ANOVA. The obtained results are given as mean ± standard variation. The comparison between groups was performed using the generalized linear model (GLM) of SAS statistical program. The multiple comparisons of means were performed using the Student Newman–Keuls (SNK) tests at a threshold of 5% (means with same letters are not significantly different, n = 3). Analysis was carried out using Xstat software (www.xstat.com).

## Results

### Bacterial identification and WGS analysis

**Fig. 1:**
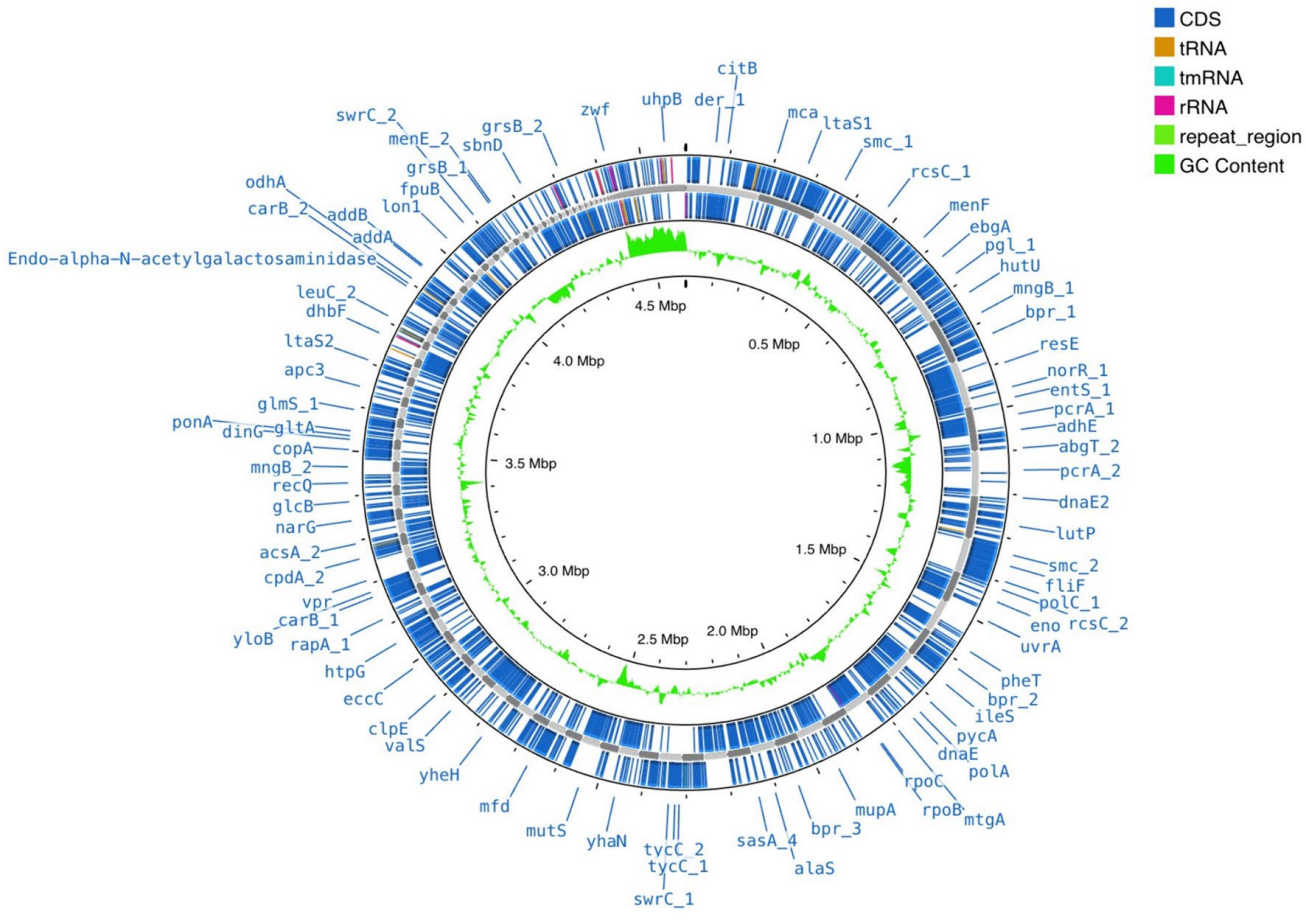
Circular genome map of the *Virgibacillus massiliensis* strain SM23 generated by CGViewer (Circular Genome Viewer) using output files from prokka annotation tool. The locations of coding DNA sequences (CDS) are shown in the outermost and second circles. The rRNA, tRNA, and mRNA genes are displayed by the third circle from the outside. The GC skew (green) is represented by the innermost circle indicating variations in DNA strand composition, often linked to replication origins. The second innermost circle represents the GC content (black), with high GC regions potentially highlighting genes involved in functions such as antibiotic resistance or stress response. Notable genes are annotated around the genome’s perimeter, including those related to metabolism (e.g., carB_2, addA), transport (e.g., glcB, narG), and stress response (smc_2, mutS). This map provides insight into the functional capabilities and adaptability of *Virgibacillus massiliensis* SM23

**Fig. 2:**
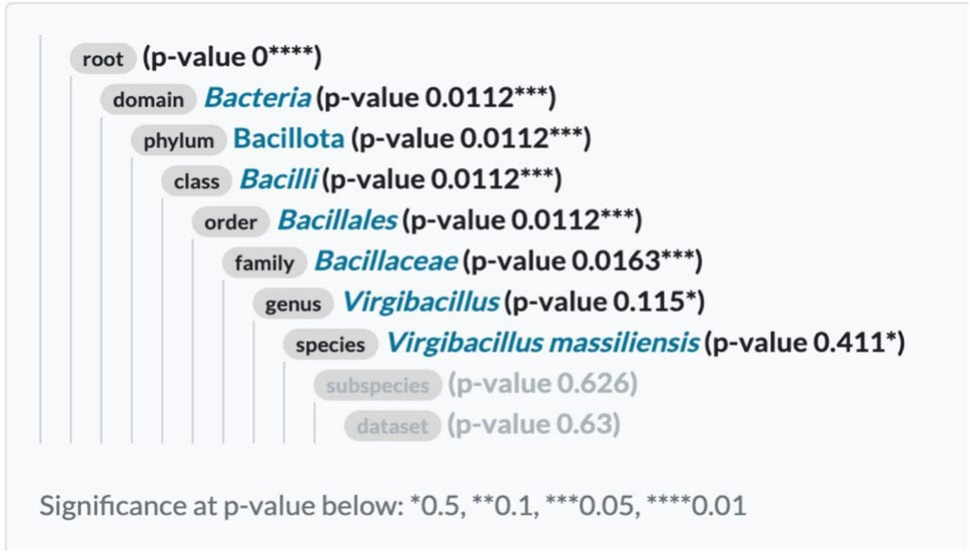
Phylogenetic placement of *Virgibacillus massiliensis* SM23, sequences blasted from Refseq Database (1865 high-quality species genomes). The classification based on the average nucleotide identity, ANI values (threshold > 95%). The bootstrap values are shown at each node

A novel bacterial strain SM-23 of *Virgibacillus* has been isolated from saline soil located in arid climate in Tunisia, to develop its biosurfactants potential by various methods. The whole genome shotgun sequencing of the bacterial strain SM-23 was conducted by Illumina using a 250 bp paired-end protocol. Genomic features of *Virgibacillus* spp. strain SM-23 are summarized in Table 1 (Essghaier et al. 2023b). Sequence reads were assembled into 127 contigs with a mean coverage of 62X and total length of 4.486,680 bp. The 16S rRNA sequence revealed a 99% sequence identity with *Virgibacillus* spp. The WGS analysis of strain SM-23 is presented in Fig. 1, more details on the genomic genes assembled are also given in the supplementary section (Additional file: Figs. S1 and S2). The classification based on the average nucleotide identity showed the phylogeny placement of the studied strain as *Virgibacillus massiliensis* (Fig. 2). Genome analysis revealed the presence of genes relevant to BSs production and indicated that these genes are highly conserved. The dataset most likely belongs to the same root of *Virgibacillus massiliensis* GCA0149054751 (*p* value: 0), probably belongs to the family *Bacillaceae* (*p* value:0.016) and possibly even belongs to the species *V. massiliensis* (*p* value; 0.41) (Fig. 2).

**Table 1:** Genomic features of *Virgibacillus* spp. strain SM-23

Bacterial strain	Strain SM-23 of *Virgibacillus spp*
Source	*Sebkhat Elmeleh*
Biosample	SAMN37231214
# contigs (**⩾**1000 bp) Largest contigs	127 185,907
Genome size (bp)	4.486.680
GC content (%)	36.65
Mean coverage	61.9538
N50	58,366
CDS	4249
tRNA genes	74
tmRNA	1
GenBank Accession (Assembly)	JAWDHA010000000
GenBank Accession (Raw reads)	SRR25870187

### Fatty acid composition of V. massiliensis SM-23

**Fig. 3:**
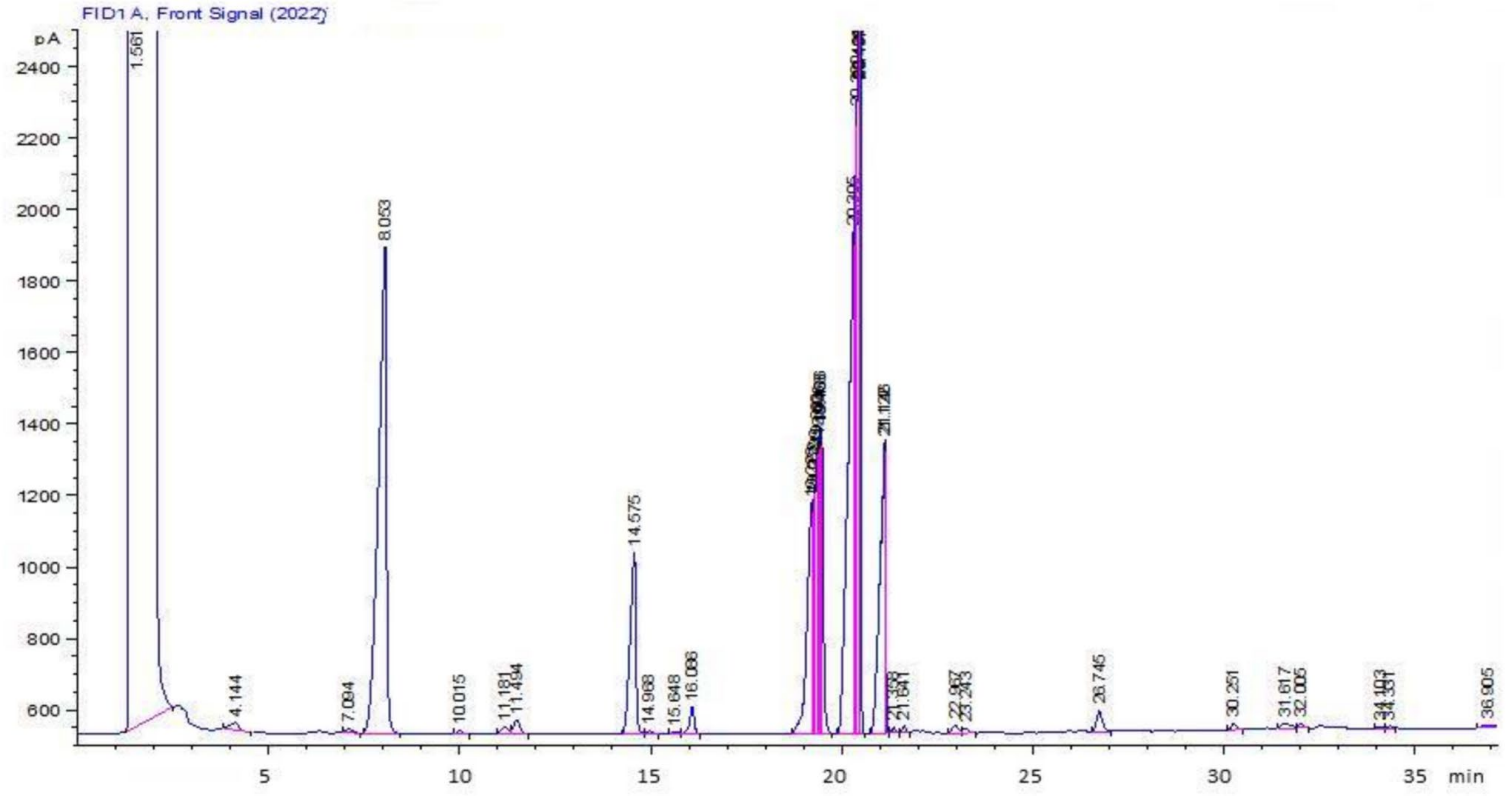
GC–MS analysis of *Virgibacillus massiliensis* SM-23 fatty acids

Because fatty acid** (**FA) biosynthesis pathway plays a crucial role in the synthesis of BSs, the total FA composition of *V. massiliensis* SM-23 was determined. The chromatogram profile of *V. massiliensis* SM-23 identified 22 FA (Fig. 3). The major FA with 33.22% was methyl 2-hyroxydodecanoate, followed by oleic acid with 23.2%, vaccenic acid (12%), methyl cis-9, 10-methylenehexadecanoate (11%), methyl 12-methyltetradecanoate (9%), stearic acid (7.08%). The other FAs range from 1.58 to 0.02% (Table 2).

**Table 2:** Fatty acid composition of *Virgibacillus massiliensis* SM-23

Fatty acid	Formula	(%)
Undecyclic acid	C11:0	0.82
Lauric acid	C12:0	0.21
Methyl.2-hyroxydodecanoate	2-OHC12:0*	**33.22**
Tridecyl acid	C13:0	0.18
Myristic acid	C14:0	0.04
Methyl. 2-hyroxytetradecanoate	2-OHC14:0*	0.83
Methyl. 3-hyroxytetradecanoate	3-OHC14:0*	0.02
Pentadecylic acid	C15:0	0.06
Methyl.13-methyltetradecanoate	iC15:0*	0.03
Methyl.12-methyltetradecanoate	a-C15:0	**9**
Palmitic acid	C16:0	0.16
Methyl.14-methylpentadecanoate	iC16:0*	0.13
Methyl.2-hyroxyhexadecanoate	2-OHC16:0*	0.05
Palmitoleic acid	C16:1n-9	0.04
Methyl.14-methylhexadecanoate	iC17:0*	**11**
Methyl cis-9,10-methylenehexadecanoate	C17:0^D^	1.58
Stearic acid	C18:0	7.08
Oleic acid	C18:1n-9	**23.2**
Vaccenic acid	C18:1n-11	**12**
Nonadecylic acid	C19:0	0.23
Methyl.cis-9,10-methyleneoctadecanoate	C19:0^D^	0.40
Arachidic acid	C20:0	0.02

Values are presented in percentage. *n–OH: hydroxyl group at C-n (n = 2,3); i: branched-chain acid with branched methyl group at the isoposition; a: branched-chain acid with branched methyl group at the anteiso position; C: carbon; D: delta. The bold numbers indicate the major components

### Biosurfactants production by bacterial strain SM-23

#### Hemolytic activity

**Fig. 4:**
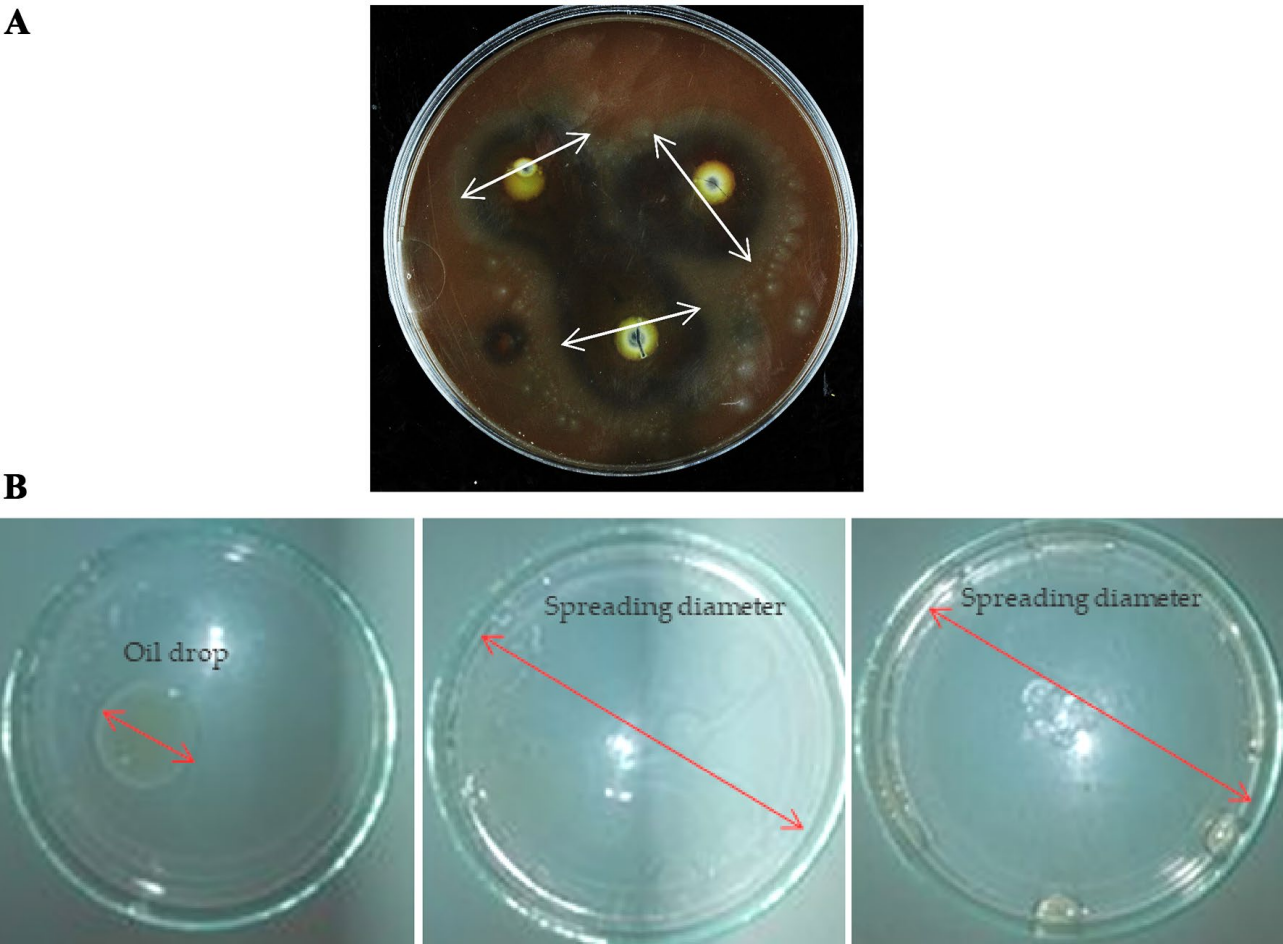
**A** The hemolysis activity of the biosurfactants produced by *Virgibacillus massiliensis* SM-23 observed on blood agar medium after 72 h. The arrow indicates the zone of hemolysis. **B** Oil spreading visualized in Petri dishes with the biosurfactants produced by *Virgibacillus massiliensis* SM-23 as compared to negative control (olive oil on distilled water), and Triton X100 as positive control

The hemolytic activity of the produced crude BSs was assayed on Blood Agar medium. The observation of a zone hemolysis with 29-mm diameter around the wells indicates the significant amount of BSs production of strain SM-23 of lipopeptides type (Fig. 4a).

#### The oil spreading test

The reduction in water–oil interfacial tension and the spreading of the oil drop were detected visually and compared to the negative control (oil drop on the water surface) and the positive control (Triton X100). The results revealed a substantial increase in spreading tension when the biosurfactants from the SM-23 strain were present, evidenced by the complete spreading of the olive oil droplet (see Fig. 4b). This characteristic is of fundamental importance as it indicates the ability of the produced biosurfactants to facilitate oil spreading.

#### The emulsification activity

**Fig. 5:**
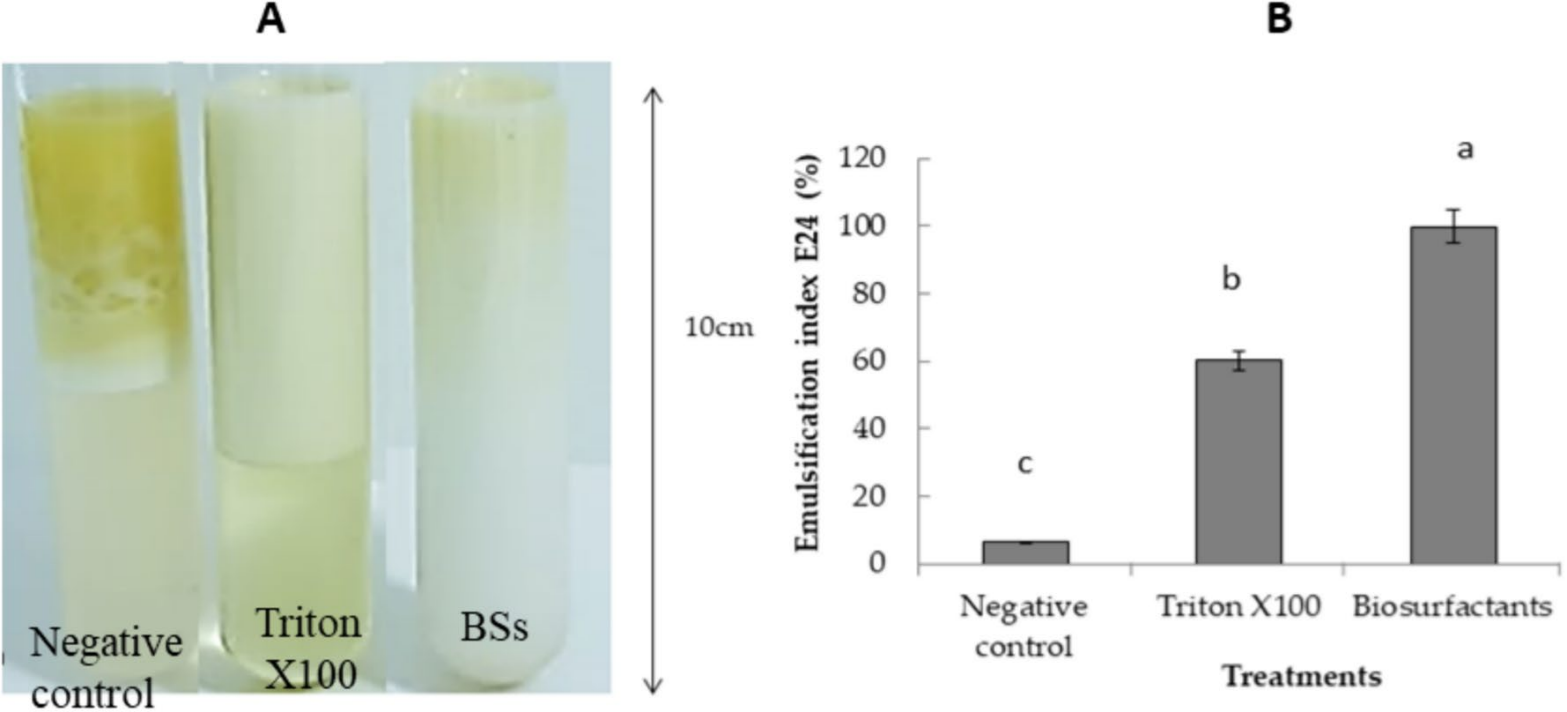
Emulsification activity (EA) of the biosurfactants produced by *V. massiliensis* SM-23 compared to the negative control and TritonX100 (**A**). The E24 index expressed in percentage as compared to the negative control and TritonX100 (**B**). Values with various letters are significantly different at *p* < 0.05

The emulsifying activity of the biosurfactants produced by the bacterial SM-23 strain was tested against olive oil on tube of 15 mm, and evaluated after 24 h. The strong emulsion expressed as a clear emulsified layer was obtained with the natural BSs produced by the strain SM-23 compared to the negative control (absence of surfactants) and Triton X100 against oil (Fig. 5A). The E24 index of emulsifying activity against oil by BSs was about 100% compared to 64% by Triton X100 and only 7% by negative control (Fig. 5B). The ES emulsification stability of BSs was calculated to be 84% after 24 h. These results indicate that the biosurfactant produced by strain SM-23 was a good emulsifier.

#### Optimal biosurfactant production

**Fig. 6:**
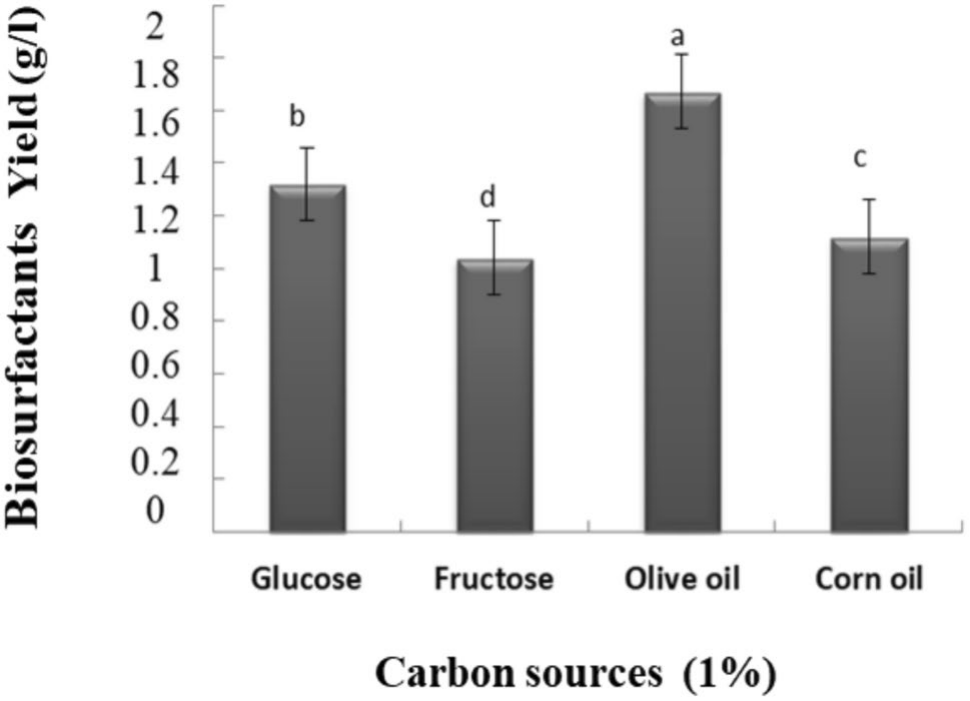
Carbon sources effect on BSs production at 30 °C. LB medium was supplemented with each of the carbon sources separately at 1%. Values with various letters are significantly different at *p* < 0.05

The carbon sources’ effects on BSs production indicated that the maximum production was obtained in LB medium supplemented with olive oil and glucose at 1%, with a crude BSs yield of 1.67 g/l and 1.32 g/l, respectively (Fig. 6). The optimal production of 1.67 g/l has been reached at 30 °C and increased to 1.92 g/l in the presence of 5% NaCl.

### Antimicrobial activity

**Fig. 7:**
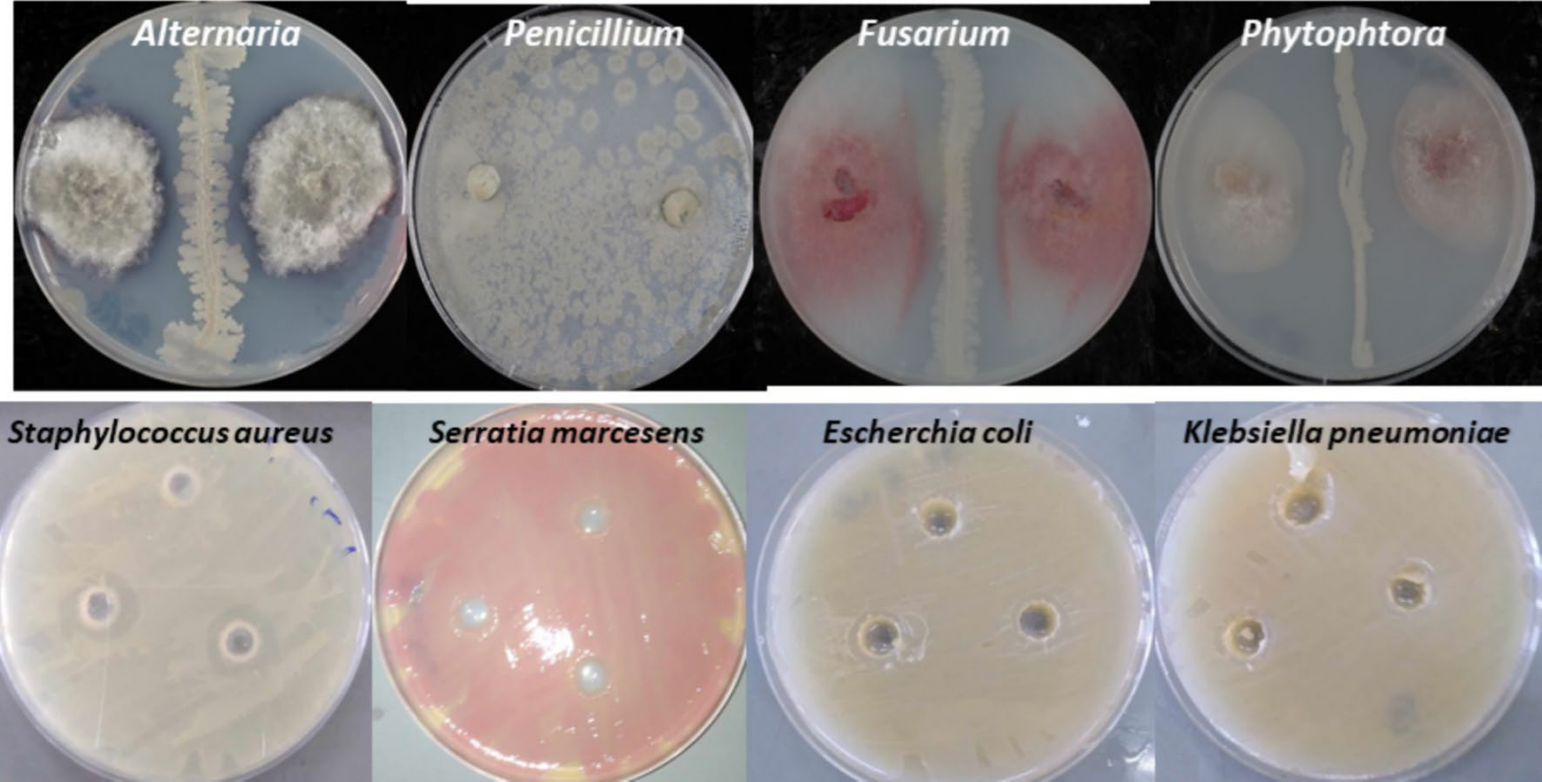
Antifungal and antibacterial actions of the strain SM-23 of *V. massiliensis*

The cell suspension of bacterial SM-23 strain showed significant antibacterial and antifungal activities against the tested bacterial and fungal pathogens, unlike they have no inhibitory effect on *Serratia marcescens* and *Penicillium* species (Table 3 and Fig. 7).

**Table 3:** Antimicrobial activities of the cell free culture of *Virgibacillus massiliensis* strain SM-23

Bacteria	Antibacterial activity	Fungi	Antifungal activity
*Escherichia coli*	+	*Fusarium*	+
*Serratia marcescens*	−	*Alternaria*	+
*Klebsiella pneumoniae*	+	*Penicillium*	−
*Staphylococcus aureus*	+	*Phytophtora*	+

(−): absence of activity, ( +): presence of activity

### FTIR analysis

**Fig. 8:**
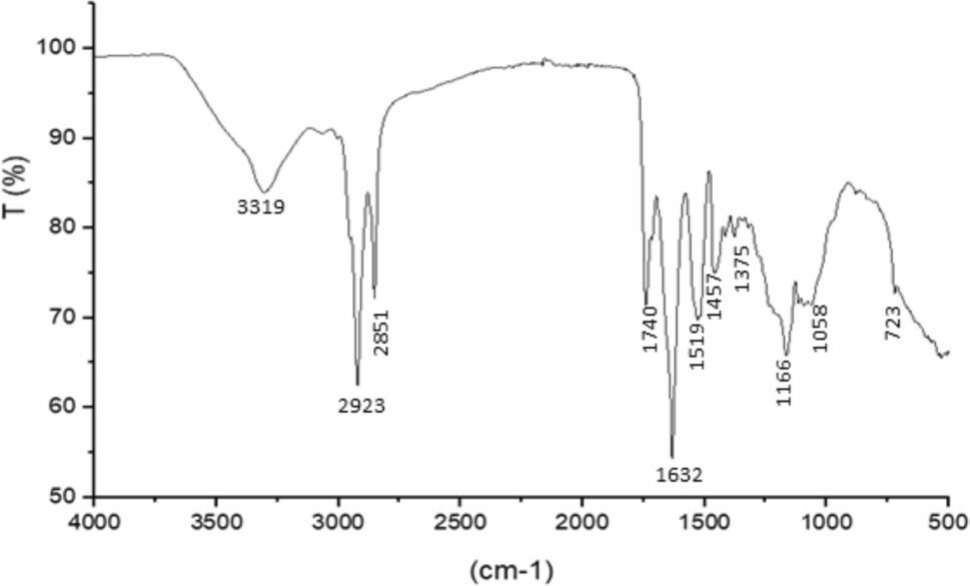
FTIR spectrum of the lipopeptides produced by *Virgibacillus massiliensis* strain SM-23

The FTIR spectrum analysis of the crude biosurfactants produced by the strain SM-23 (Fig. 8) showed bands characteristics of peptides at 3319 cm^−1^, which correspond to NH-stretching mode and band at 1632 cm^−1^ correspond to C–O stretching and N stretching, a characteristic of amino group in cyclic lipopeptides. The band at 2923 cm^−1^ indicates the methyl group. The bands at 2851 cm^−1^, 1457 cm-^1^, and 1375 cm^−1^ indicate the presence of aliphatic chain (CH3, CH2). High peak observed in the region 1000–1100 cm^−1^ was assigned to O–C–O extend vibrations of carboxylic acids, aldehydes, and ketones, respectively, which indicate the oxidations of hydroxyl groups of hydrolysates from medium peptides.

### Analysis of secondary metabolite biosynthetic gene clusters

**Fig. 9:**
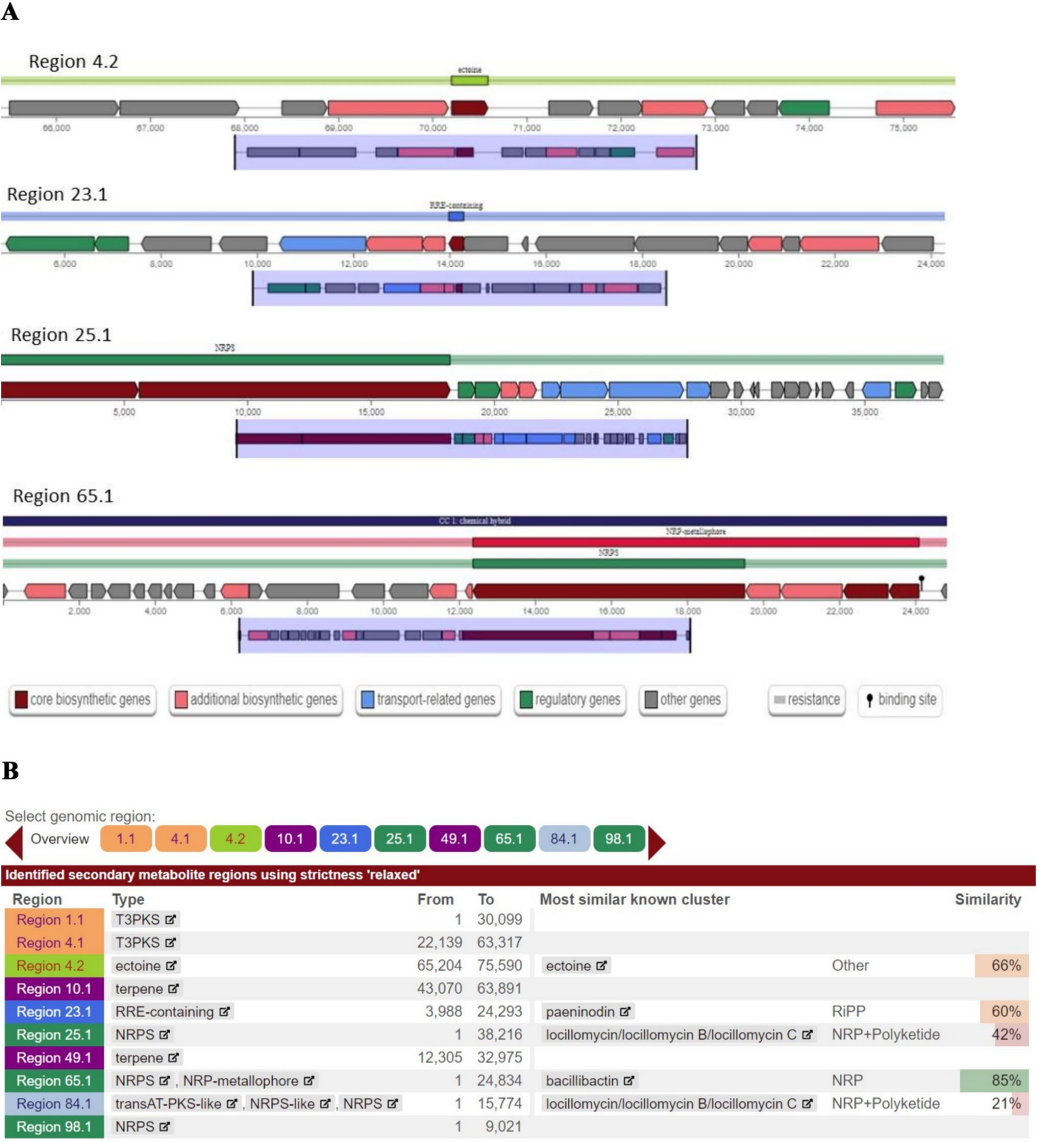
**A** Secondary metabolic biosynthetic gene cluster analysis of the bacterial strain SM-23 detected by antiSMASH bacterial 7.1.0 using a specific profile hidden Markov models. **B** Summary of the genomic regions including the secondary metabolites regions and the identification of the most Biosynthetic Gene Clusters (BGCs) involved in the biosurfactants metabolism production by antiSMASH bacterial 7.1.0

Basic gene findings of the strain SM-23 genomic sequence indicated that 4486680pb genome size are predicted and classified into 576 nodes; among these, a number of 231,299 functional genes are involved in the secondary metabolites biosynthetic, transport and catabolism. AntiSMASH analysis suggested that the SM-23 bacterial strain possesses six secondary metabolites biosynthetic gene clusters (BGCs), with biotechnological interest, including two type III PKSs (T3PKSs), three NRPSs, one NRPS-metallophore, one ectoine, one RRE-containing and two terpene genes (Fig. 9B). The PRE recognition element is a widespread domain in various classes of peptides ribosomally synthesized and post-translationally modified RiPPs (Kloosterman et al. 2020).

Based on the MIBiG database, our findings indicate that only 50% of BGCs exhibit gene homologies with known clusters. Several BGCs of the bacterial strain SM-23 were predicted to be intervening in the biosynthesis of bacillibactin in the region 65.1, ectoine in the region 4.2, paeninodin in the region 23.1, and locillomycin/locillomycin B/locillomycin C in the region 25.1 and 84.1 (Fig. 9A).

**Fig. 10:**
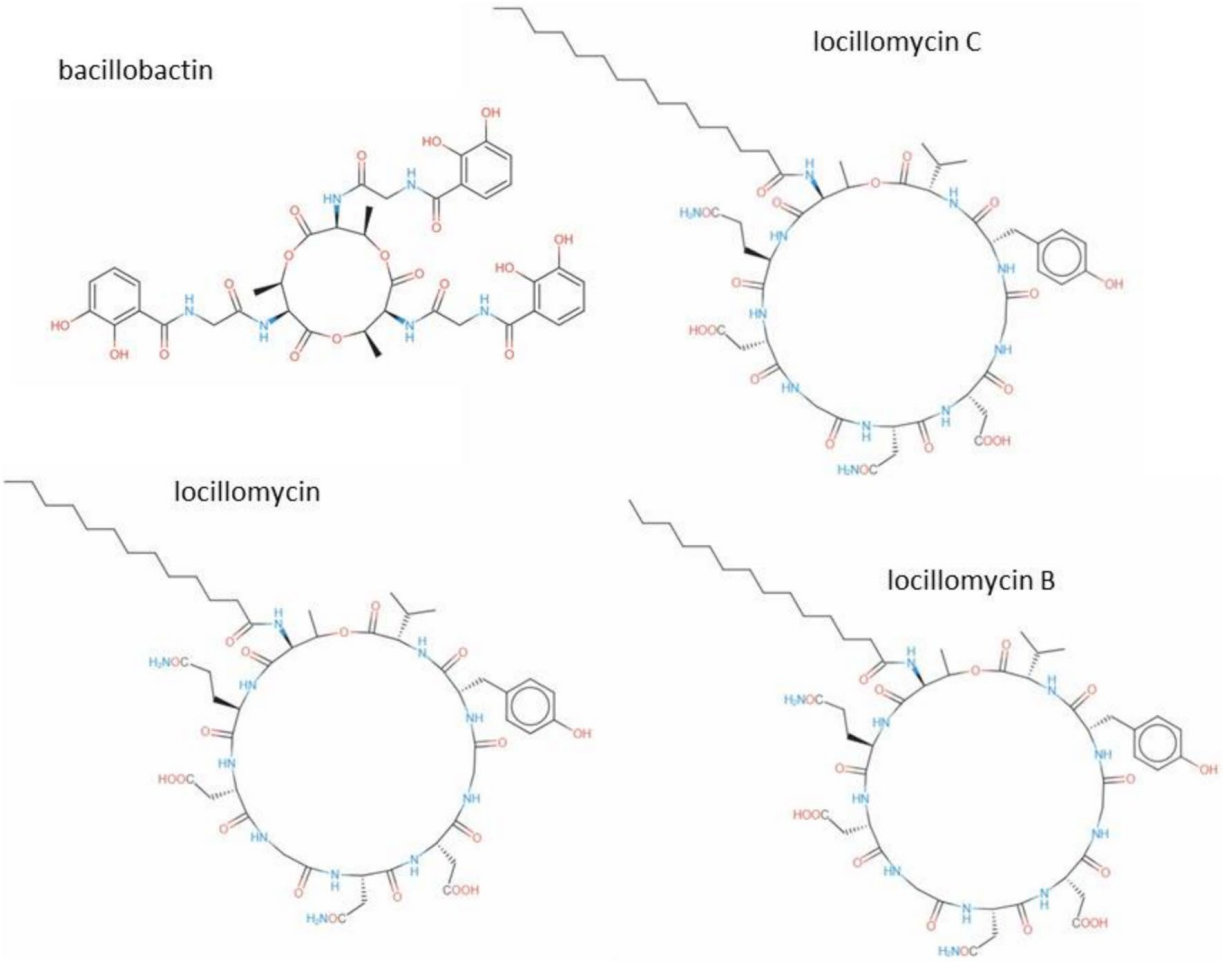
The predicted structure of the cyclic lipopeptides produced by the strain SM-23 of *Virgibacillus massiliensis*

AntiSMASH analysis showed that the genes within the region 4.2 had a significant BLAST hit with the ectoine BGC (GenBank: AF316874.1) from *Sporosarcina pasteurii.* While BGC region 23.1 of strain SM-23 displayed significant similarity with those of paeninodin from *Paenibacillus dendritiformis* C454 (GenBank: AHKH01000064.1). Paeninodin belonging to lasso peptides are a new class of RiPPs, which are isolated from proteo- and actinobacteria. In addition, the non-ribosomal peptide synthetase BGC in the region 25.1 of the strain SM-23 showed a similar sequence to the locillomycin/locillomycin B/locillomycin C (GenBank: KF866134.1) from *Bacillus subtilis.* The predicted chemical structure of locillomycin/locillomycin B/locillomycin C and bacillibactin is presented in Fig. 10. In contrast, for Paeninodin, there is no structure information available in antiSMASH bacterial 7.1.0. Non-ribosomal peptides produced (NRPSs) involved in the biosynthesis of bacillibactin were highly similar (with similarities > 80%) to the BGC of bacillibactin BGC from *Bacillus subtilis* subsp. *subtilis* str. 168 (GenBank AL009126.3).

## Discussion

Biosurfactants (BSs) offer numerous advantages over synthetic counterparts, including enhanced bioavailability, structural diversity, and specific activity even under extreme conditions of temperature, pH, and salinity (Zhang et al. 2022; Leoni et al. 2022). In this study, we aimed to isolate a novel bacterial strain SM-23 from the extreme environment of Sebkha ELMeleh in Tunisia, with the potential to produce biosurfactants. To our knowledge, this work represents the first description of BSs sourced from the *Virgibacillus massiliensis* species. Assessing the chemical structures and biological properties of microbial BSs, along with considering the relative costs of production, are crucial parameters in selecting microorganisms as BSs producers. In addition, factors such as the type of substrate and purification strategy play vital roles in this selection process (ErasMuñoz et al. 2022). The high limitations of biosurfactants in biotechnological industries are the yield production and the high costs, in this regard, It should be noted that in terms of biosurfactants yield production, the described strain SM-23 of *V*. *massiliensis* synthesizes more higher amount of lipopeptides (with 1.62 g/l), compared to other *Bacillus* species such as *Bacillus subtilis* strain with 0.545 g/l after optimization approach of culture. After the complex approach of overexpression of genes to obtain a recombinant higher producer strain of *Bacillus subtilis*, it can only produce similar yields of surfactin (1.67 g/l) (Nawazish et al. 2022).

Microbial biosurfactants (BSs) are excreted onto the microbial cell surface and then released into the extracellular environment. Cyclic lipopeptides from the *Bacillus* genus, such as bacillomycin, fengycins, and surfactin, have been extensively studied (Giri et al. 2019; Nawazish et al. 2022). Given the wide applicability of most *Bacillus* lipopeptides in industries such as food, cosmetics, and pharmaceuticals, numerous studies have focused on optimizing their production by altering carbon sources. Strain SM-23 of *V. massiliensis* exhibits maximum BSs yield when olive oil is utilized as a carbon source. BSs can be synthesized from various substrates, including renewable resources, vegetable oils, and distillery dairy wastes. Maximum rhamnolipid production has also been demonstrated by *Pseudomonas* in the presence of olive oil (Ji et al. 2016). Our current study confirms that BSs production can indeed be enhanced by employing olive oil as an inducer substrate. In the present work, we first combine the BSs production and WGS information from *V. massiliensis*, to identify the gene cluster involved in the BSs production in the studied conditions. The analysis of the genome context of dpp transporter of dipeptides and short peptides, of strain SM-23, reveals the presence of genes oppB (2 copies), oppD (4 copies), oppF (3 copies), dppA (1 copy), dppB (2 copies), dppC (2 copies), dppE (4 copies), as compared to the genome of *Bacillus subtilis*, gene dpp and opp were also conserved (Hughes et al. 2022). In fact, these genes of transporter play important role in nutritional and cell signaling in *Bacillus*. Several genes involved in lipid metabolism were also conserved in the genome of strain SM-23 of *V. massiliensis* such as genes lapA, tmpC COG, murJ, IcfB, gene lipA, fadR, and fad D, encoding to the FA metabolism regulator protein, LPS assembly protein A, membrane lipoprotein TmpC, fatty acid-binding protein TM_1468, lipid II flippase MurJ, Lipoyl synthase long-chain-fatty-acid-–CoA ligase, long-chain-fatty-acid–CoA ligase, respectively. These findings confirm the production of various lipopeptides detected in the genome of the described strain SM-23.

In the context of fatty acid (FA) metabolism, all microorganisms utilize acetyl Co-A as a substrate for synthesizing straight-chain FAs (SCFAs), while branched-chain fatty acids (BCFAs) stem from isobutyryl CoA and isovaleryl CoA. BCFAs constitute the primary components of phospholipids, with iso C15:0, anteiso C15:0, iso-C16:0, iso-C17:0, and anteiso-C17:0 identified as major fatty acids found in Bacillus species. Environmental conditions can influence the composition of BCFAs. Literature suggests that the FA biosynthesis pathway plays a crucial role in BSs synthesis, with modifications to FA composition (such as length and isomerism) impacting the physicochemical and biological properties of lipopeptides. Consequently, several authors have endeavored to enhance lipopeptide-producing bacteria, as exemplified by studies of Geissler et al. (2019) and Dhali et al. (2017).

Regarding the FA composition among other *Virgibacillus* species, the results illustrate that the FA profile is related to each *Virgibacillus* species, for example: C15:0 anteiso was also detected in the FA profile of *V. doumboii, V. siamensis* and *V. pantothenticus* with 49%, 55.8%, and 47.4%, respectively. Unlike strain SM-23 of *V. massiliensis* shows only 9% C16:0 and C14:0 varying from *Virgibacillus* species to others. C17:0 iso was also detected in FA of *V. siamensis* strain JCM15395 and *V. pantothenticus.* These findings affirm that the composition of fatty acid profiles varies among species and are influenced by the composition of the culture medium, as demonstrated by Konate et al. (2021).

Strain SM-23 shows 22 FA, and the major FA are methyl.2-hyroxydodecanoate, BCFAs with a methyl chain branching point in the middle of the hydrocarbon chain that are specific to some bacteria (Gunstone and Harwood 2007). Several research projects focused on the occurrence and benefits of BCFAs (Gozdzik et al. 2023; Gozdzik et al. 2023; Lu et al. 2024; Yang et al. 2023). Thus, further studies needed to understand the benefits of BCFA produced by our bacterial strain as a high producer BSs.

Similar results of the abundance of methyl 2-hydroxydodecanoate and oleic acid in fatty acid composition of the bacterial cell membrane were also obtained in the membrane of halotolerant specie *Pantoea alhagi* (Essghaier et al. 2023a). In literature, several reports mentioned that membrane lipid and the FA associated are influenced by the environmental conditions (Hartmann-Balogh et al. 2022). Fatty acid content affects fluidity, flexibility, and permeability of the cell membrane (Cronan and Luk 2022). Biotransformation of fatty acid methyl esters to dicarboxylic acids has gained the attention of several research projects; decane induction has been reported to be essential to activate the enzyme pathway (Sugiharto et al. 2018).

Moreover, ensuring optimal nutritional properties is essential for formulating cost-effective production of biosurfactants (BSs) (Nurfarahin et al. 2018). In this study, we present preliminary results on the impact of carbon sources, such as a vegetable oil, on BSs yield. However, further investigations are warranted to optimize production, including exploration of alternative carbon and nitrogen sources, as well as consideration of macro-nutrient availability and the carbon/nitrogen ratio. The secretion of microbial BSs is intricately linked to the metabolic pathways associated with carbon sources present in the culture medium, particularly hydrophobic substrates (Nurfarahin et al. 2018; Desai and Banat 1997). Microbial metabolism is profoundly influenced by the availability of carbon sources, which in turn regulates both lipogenic and glycolytic pathways (Haritash and Kaushik 2009).

Several researchers have demonstrated the efficacy of hemolysis tests, oil spreading tests, and emulsification assays for detecting BSs (Essghaier et al. 2023a). Lipopeptides exhibit hemolytic activity, with examples such as those derived from *Bacillus* species capable of inducing hemolysis in human erythrocytes (Gurkok 2022). The results obtained, including a higher zone of lysis on blood agar medium (28 mm) and an E24 Index of 100%, compared favorably to other BSs reported from *Bacillus* species, demonstrating the superior performance of the novel isolate described in this study as a high producer of BSs. To our understanding, among the *Virgibacillus* genus, only BSs produced by *Virgibacillus salarius* have been published. Similar methods, including drop collapse assays, oil displacement tests, blood hemolysis assays, emulsification activity assessments, and surface tension measurements, were employed to evaluate BS secretion from *V. salarius* (Elzzazy et al. 2015).

The obtained results show the stability of BS production by the strain SM-23 of *Virgibacillus massiliensis* at the range of temperature and salinity; similar data on BSs stability were reported by *Virgibacillus salarius* (Elzzazy et al. 2015). Unlike, the BSs produced by *V. salarius* show its emulsification activity (E24) of about 80% compared to E24 of 100% given by the BSs of *V. salarius* (Elzzazy et al. 2015). In addition, *V. salarius* can produce similar BSs yield with NaCl ranging from 0 to 10% with a maximum at 4% of 1.6 g/l and 1.64 g/l at 40 °C, at pH9 BSs of about 1.51 g/l (Elzzazy et al. 2015). Unlike our strain SM-23 of V. *massiliensis* shows more significant BSs production with 1.67 g/l at 30 °C and 1.92 g/l in the presence of 5%NaCl. Thus, these results show markedly significant production of BSs by the strain SM-23, and encourage its use as a microbial producer for the commercial production of BSs.

FTIR analysis revealed the presence of functional groups, indicating that the biosurfactants produced by the SM-23 strain were lipopeptides, as evidenced by the NH stretching group corresponding to the peptide component’s aliphatic chains. Unlike glycolipids, the *Bacillus* genus synthesizes heterogeneous lipopeptides with varying structures. The structure of *Bacillus* lipopeptides is characterized by differences in peptide residues, fatty acid chain branching, and length (Guo et al. 2015). This finding parallels the work of Elzzazy et al. (2015), who demonstrated the production of lipopeptides by *Virgibacillus salarius*. In addition, the FTIR analysis shows high similarity to cyclic lipopeptides like surfactin and Bacillibactin produced by *Bacillus subtilis* as previously reported (Jha and Joshi 2016). FTIR spectrum data confirm that the biosurfactants produced by strain SM-23 belong to cyclic lipopeptides NRPSs as illustrated in the genome showing high similarity of 80% with bacillibactin from *Bacillus subtilis*.

The lipopeptides produced by strain SM-23 show antibacterial and antifungal activities against tested microbial pathogens. It is widely recognized that microbial biosurfactants possess antibacterial, antifungal, and antiviral properties (Naughton et al. 2019). Surfactin demonstrates hemolytic, antiviral, and antibacterial properties; however, its antifungal activity is comparatively limited (Maksimova et al. 2020). It is well documented that iturin and fengycin produced by *Bacillus* species show antifungal action against *Aspergillus flavus* and *Fusarium* (Nawazish et al.^2022^); however, the new strain SM-23 of *Virgibacillus massiliensis* produced different lipopeptides with strong antibacterial and antifungal actions against all tested pathogens.

The genome analysis of strain SM-23 revolved the presence of BGCs with high similarity identified to be responsible for the biosynthesis of ectoine, bacillibactin, paeninodin, and locillomycin/ locillomycin B/locillomycin C. Ectoine as a highly water keeping compound stabilizing biomolecules and whole cells can be used in scientific work, cosmetics, and medicine (Alexander et al. 2011). Lasso peptides are the most important class of RiPPs exhibiting antimicrobial, antiviral, receptor antagonistic or enzyme inhibitory activities (Zhu et al. 2016; Wang et al. 2021). Among the non-ribosomal generated amphipathic cyclic lipopeptides, locillomycin/locillomycin B/locillomycin C are well-recognized by their potential applications in biotechnology and biopharmaceutical products due to their antagonistic activities and surfactant properties. Bacillibactin have catecholic siderophores in iron acquisition (May et al. 2001; Abi Khalil et al. 2014). Bacillibactin class of siderophores antibiotics show antibacterial properties (Chakraborty et al. 2022).

Regarding the BSs production, there are three methods to consider: utilizing crude extracts, pure biosurfactants, or biosurfactant-producing microorganisms. Our research advocates for the use of biosurfactant-producing microorganisms. However, comparing these methods is crucial before considering future applications to optimize BSs activity and stability. Thus, the work presents a preliminary study aimed to describe new high BSs producer bacteria. The current finding confirms that BSs pathways are related to the carbon sources and environmental conditions; therefore, as continuity of this research, we will study the metabolic pathway of the strain SM-23 to enhance the lipopeptides production. On the other hand, the action of the signaling protein NRP-1 in inducing the jasmonate-ethylene signaling pathway to enhance the plant immune system (Maksimova et al. 2020), along with the presence of multiple lipopeptides compounds produced by *V. massiliensis* SM-23, with its significant antifungal action, suggests the opportunity to utilize these lipopeptides in the formulation of biopesticides to protect plants from phytopathogenic fungi.

## Conclusion

The novel discovered bacterial strain *Virgibacillus massiliensis* SM-23 has demonstrated the ability to produce various lipopeptides identified through WGS analysis. The production of these lipopeptides has been confirmed through various methods. Compared to other species, the strain SM-23 produces lipopeptides under the tested conditions LB medium with 1% olive oil and 4% NaCl at 30 °C, which exhibits significant activity. These preliminary results could contribute to improving the production of biosurfactants by strain SM-23, particularly through the stimulation of lipopeptides metabolic pathways. This current study suggests that strain SM-23 could be a promising candidate for the development of natural antimicrobial drugs. Finally, the produced lipopeptides with low toxicity and efficient biodegradability stimulate their use for the bio-remediation process of organic and inorganic pollutants.

## Supplementary Information

Below is the link to the electronic supplementary material.

Supplementary file1 (DOC 328 kb)

## Data Availability

The Whole Genome Shotgun project has been deposited at DDBJ/ENA/GenBank under the accession JAWDHA000000000. The version described in this paper is version JAWDHA010000000. Raw sequence reads have been deposited in the NCBI Sequence Read Archive SRR25870187, under BioProject number PRJNA1011906.
