# Supplementary material for: Discovery and characterization of novel lipopeptides produced by Virgibacillus massiliensis with biosurfactant and antimicrobial activities
Source: 3 Biotech. 2024 Oct 4;14(11):258. doi: 10.1007/s13205-024-04100-9 (PMC11452367; doi:10.1007/s13205-024-04100-9)
Supplement: Supplementary file 1 — Supplementary file1 (DOC 328 kb) [file 13205_2024_4100_MOESM1_ESM.doc]

Additional file: Fig. S1 Fig. S2


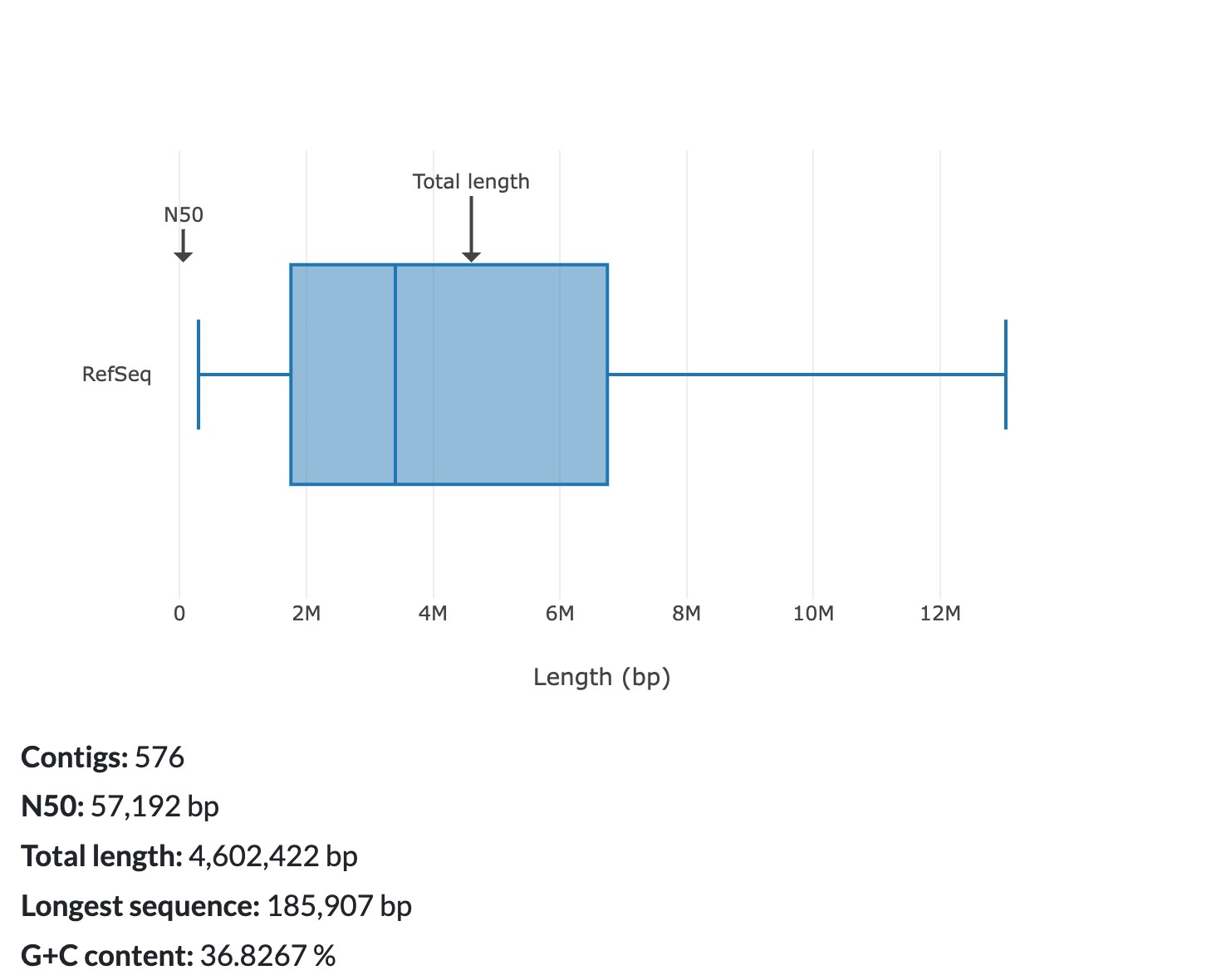


**Fig. S1** Quality assessment of contigs based on QUAST (nb of contigs, N50, Total length, Longest sequence, CG content ...)


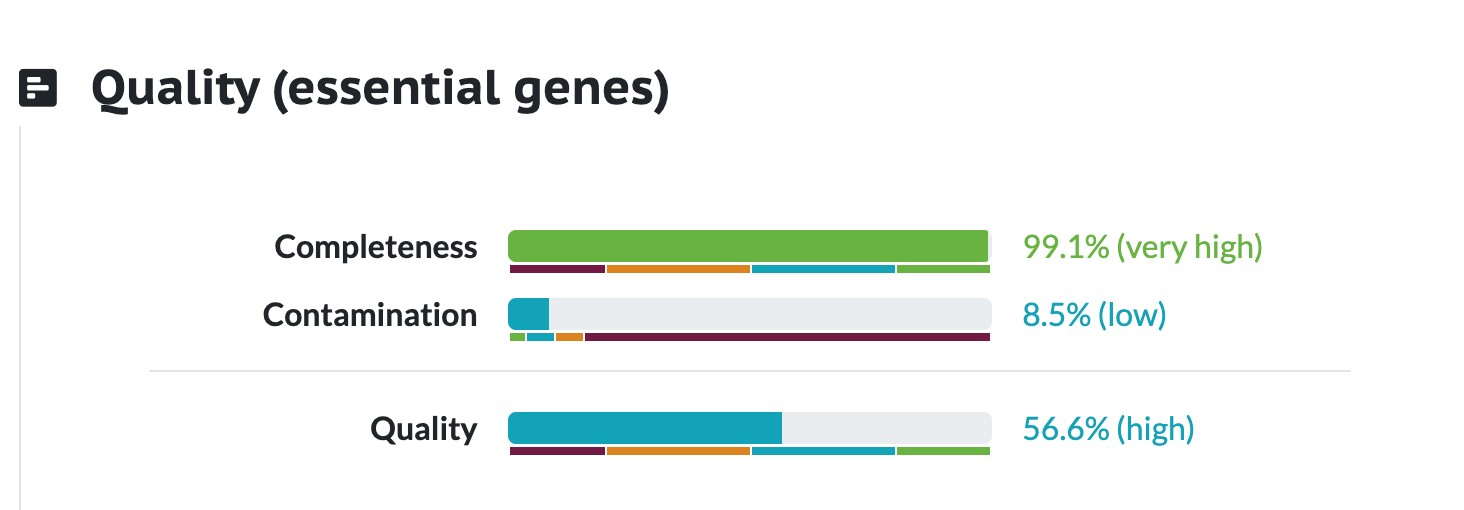


**Fig.S2** Quality assessment of assembled genomes based on CheckM (completness, quality, contamination)
